# Limitations of point-of-care testing for low SARS CoV-2 loads: Insights for future pandemics

**DOI:** 10.4102/phcfm.v17i1.4671

**Published:** 2025-03-20

**Authors:** Ivy Rukasha

**Affiliations:** 1 Department of Pathology, Faculty of Health Sciences, University of Limpopo, Polokwane, South Africa; 2 Department of Medical Microbiology, National Health Laboratory Services, Polokwane, South Africa

**Keywords:** poor performance, point-of-care tests, COVID-19, low viral load, antigen tests, diagnostic accuracy, SARS CoV-2

## Abstract

**Background:**

The emergence of severe acute respiratory syndrome coronavirus 2 (SARS-CoV-2) has seen a surge in the development of diagnostic assays. However, the performance of antigen point-of-care tests (Ag-POCTs) on samples with low viral load has not been evaluated.

**Aim:**

To evaluate the accuracy of three World Health Organization (WHO) certified Ag-POCTs in comparison to the reverse transcription polymerase chain reaction (RT-PCR) technique.

**Setting:**

The study was conducted at Pietersburg Hospital Limpopo, South Africa between March 2020 and April 2023.

**Methods:**

A total of 371 SARS-CoV-2 nasopharyngeal samples from the National Health Laboratory Service were tested using Ag-POCTs from Abbott Panbio, Roche RDT and SD Biosensor, following manufacturer instructions. All samples had RT-PCR results with Ct values between 13 and 45. Reverse transcription polymerase chain reaction results were compared and correlated with Ag-POCT results.

**Results:**

Of the 371 samples, the SD Biosensor Standard Q test kit detected the most positive isolates 166 (44.7%), followed by the Abbott Panbio. A total of 153 (41.2%) positives, while the Roche SD detected 134 (36.1%) samples. High viral load (Ct < 25) sensitivity and specificity exceeded 77%, while intermediate (Ct 25–35) and low viral load (Ct > 35) sensitivity and sensitivity dropped to 32% and 7%, respectively.

**Conclusion:**

The performance rapid antigen tests was low on samples with low viral load with results markedly different from the manufacturer’s reported performance.

**Contribution:**

Rapid antigen tests should not be used alone for diagnosis, especially in samples with low viral load.

## Introduction

The coronavirus disease 2019 (COVID-19) outbreak exposed a major weakness in the laboratory’s capacity to deliver results when faced with a global pandemic, particularly in low-resourced regions. Developing countries’ laboratories were left with no answer as the pandemic ravaged the society.^1^ The unprecedented pressure has almost put the laboratory network on the verge of collapse in many developing countries. As a response to the pandemic, there has been a surge in the number of point-of-care tests (POCs) which were developed and made commercially available because of the critical demand for alternative diagnostic assays during the pandemic.^1^

The gold standard and reference procedure for diagnosis of severe acute respiratory syndrome coronavirus 2 (SARS-CoV-2) infection is the reverse-transcriptase polymerase chain reaction (RT-PCR). The RT-PCR assays typically amplify and detect viral components which include genes of the four structural proteins of SARS-CoV-2 structure, namely spike (S) and membrane (M) glycoproteins, as well as envelope (E) and nucleocapsid (N) proteins. The RT-PCR has a high specificity of around 100% (95% CI: 98.9% – 100%) and a sensitivity of 80.7% (95% CI: 73.2% – 86.9).^2^

However, despite the RT-PCR’s unmatched precision and accuracy, the assays had many disadvantages, such as the need for specialised equipment, highly trained staff, laboratory equipment, and/or the high cost and time necessary per test.^3^ As a result, specimens had to be transported to central facilities with RT-PCR capability, delaying test outcomes and thus results that may be clinically irrelevant among suspected COVID-19 patients.^4^

Immunoassays offer an alternative to molecular tests.^5,6^ The COVID-19 point-of-care antigen tests (Ag-POCTs) provide alternative assays to diagnose and screen active infection by detecting SARS-CoV-2 viral proteins in various specimen types.^5^ Point-of-care tests have the advantage of providing real-time results within 20 min that help in making prompt clinical decisions and reduce follow-up visits or calls, require comparatively less sample volume to tests, lower costs than PCR, and greater access to testing of SARS-CoV-2 particularly in areas further away from PCR labs.^6^

Three types of POCs have been developed during the pandemic namely, molecular, antibody and antigen-based assays.^7^ Molecular-based POC technologies require equipment in the form of hand-held readers, mobile laboratory, and reaction–based Bench top systems while antibody-based assays may not confirm the presence of active viruses because of their diagnostic limitations in early infections.^7^ Antibodies are produced at a later stage of infection approximately after 15 and more days after exposure to the SARS-CoV-2 infection. Thus, antigen assay POCs have become the preferred POC.^8^

Low-resourced areas such as Limpopo Province have completely switched from lab-based assays to use rapid tests to diagnose COVID-19.^8^ However, the performance of these POCs in patients with low-viral load has not been assessed. During the course of infection, SARS-CoV-2 detection is often missed by Ag-POCTs because of low-viral load in clinical specimens in asymptomatic and/or recovery phases, whereas the patient could be a potential super-spreader of COVID-19.^9^ Accuracy of POC performance has a direct bearing in perpetuating diseases and the development of new infections. Thus, this study was conducted to evaluate the diagnostic accuracy of three Ag-POCTs in comparison to the RT-PCR technique on samples with low viral load from the Limpopo Province population during the pandemic era.

## Research methods and design

### Study design

The study was a descriptive cross-sectional design, which involved retrospectively testing nasopharyngeal specimens for SARS-CoV-2. These samples had been collected from patients and stored between March 2020 and April 2023.

### Study setting

The study was a descriptive cross-sectional research conducted at the Pietersburg Hospital, Limpopo Provincial Hospital, Polokwane, South Africa. The hospital is the only referral tertiary hospital for the fifth largest Province of South Africa serving around 5.5 million people and has a 500-bed capacity. The province is situated in northern South Africa bordering Mozambique, Zimbabwe and Botswana. Limpopo is one of South Africa’s poorest provinces and is predominantly rural (80%) with relatively inadequate infrastructure and public services, including healthcare. The province is pivotal to South Africa disease trends as it serves as a major road gateway between South Africa and other African nations north.^10^

### Study population and sampling

The study population consisted of all patients in the Limpopo Province, South Africa who have samples collected for SARS-CoV-2 testing, based on clinical grounds. These samples were sent to the National Health Laboratory Services Lab at Pietersburg Hospital. The study involved convenient sampling that included all available nasopharyngeal samples that met inclusion criteria that were sent to the National Health Laboratory Service (NHLS). Nasopharyngeal specimens were collected from patients suspected of having COVID-19. Convenience sampling will mean including residual nasopharyngeal and nasal samples obtained from virology lab from NHLS Polokwane Laboratory repository. The samples were stored at –40° C from March 2020 to April 2023. A total of 500 nasopharyngeal swabs were initially included. Duplicate samples and those with missing demographic information (age and gender) were excluded from analysis. The samples were categorised based on Cycle threshold (Ct) values from RT-PCR into: Low viral load (Ct ≥ 35), Intermediate viral load (25 ≤ Ct < 35) and High viral load (Ct < 25). This structure provided a diverse representation of viral loads within the study population, which was crucial for evaluating the performance of the Ag-POCTs against the RT-PCR technique.

### Data collection

The study obtained retrospectively tested nasopharyngeal specimens RT-PCR results. The remnant samples had been stored at -40° C for varied periods ranging from March 2020 to April 2023. All stored nasopharyngeal samples were obtained from patients suspected of COVID-19 whose samples were sent to the virology laboratory for routine analysis. The demography of the patient was recorded from the laboratory request forms submitted with the samples. All samples included in the study had RT-PCR results and Ct values obtained from prior routine patient analysis.

### Real-time polymerase chain reaction assay procedure

Primarily SARS-CoV-2 RT-PCR testing was performed as part of routine testing at the Polokwane NHLS Virology laboratory. The SARS-CoV-2 RT-PCR was performed using either of the Cepheid GeneXpert® Xpert Xpress SARS-CoV-2 assay (Sunnyvale, California, United States). In general, the RT-PCR assay is based on a real-time RT-PCR technique used to qualitatively detect SARS-CoV-2 RNA in nasopharyngeal swabs, nasal swabs and nasal aspirate specimens. Information obtained from the RT-PCR included categorical results of RT-PCR SARS-CoV-2 testing along with their Ct values.

### Antigen bases assay procedure

The remaining nasopharyngeal swab samples were subjected to three antigen tests namely the Abbott Panbio COVID-19 Ag Rapid Test Kit (Sunnyvale, California, United States), the Roche SD Biosensor rapid antigen test (Roche Diagnostics, Switzerland), and the standard SD Biosensor Antigen Q test (SD Biosensor, South Korea).

All Ag-PoCT test kits were solid-phase immuno-chromatographic assays used for the qualitative detection of SARS-CoV-2 samples. Tests were performed according to the manufacturer’s instructions. The test kit contains colloidal gold conjugated with recombinant COVID-19 antigen (COVID-19 antigen) (COVID-19 conjugate). When the assay buffer sample is added to the sample well, if antibodies are present, they will bind to the COVID-19 conjugate to form an antigen-antibody complex. This complex passes through the nitrocellulose membrane by capillary action. When the complex meets the line of the corresponding stationary object, it is captured and forms a burgundy band, confirming the positive. If there is no coloured band in the test area, the test result is considered negative. Two trained technicians independently interpreted each result. When reading the assay, the technicians were blinded to the results of other assays as well as to each other’s results. Discordant readings between the two technicians on results were arbitrated by consultation with a third technician.

### Data analysis

A total of 500 nasopharyngeal swabs were initially included in the study. Unique identifiers that included laboratory episode numbers and their date of birth were used to remove duplicate samples. A total of 90 samples were found to be duplicates while 39 samples were found to have missing demographic information age and gender and thus removed from the study. Thus, the study ultimately included 371 samples, whereby 291 (78.4%) were RT-PCR positive and 80 (21.6%) were RT-PCR negative.

For analysis, the samples were categorised into three groups namely, low viral load consisting of Ct ≥ 35, intermediate viral load consisting of 25 ≤ Ct < 35, and high viral load constituting of Ct < 25. The relationship between Ct values and viral load quantity is inversely proportional, thus, samples with high viral load had SARS-CoV-2 RNA amplified earlier on RT-PCR hence lower Ct values, and vice versa. There were 156 (53.6%) samples belonging to the low viral load category, 83 (28.5%) samples belonging to the intermediate viral load category, and 52 (17.9%) samples in the high viral load category. Lastly, test performance was analysed using the SPSS version 27.0 software. Performance indices (sensitivity, specificity and accuracy) per Ag-POCT were determined for each category.

A total of 371 specimens were included for further analysis and imported to SPSS version 27.0. Categorical variables were described as numbers and percentages. Figures were used to show the pictorial presentation of the data. Descriptive statistics were used, and continuous variables were expressed as median and interquartile range.

### Ethical considerations

Ethical clearance to conduct this study was obtained from the University of Limpopo Turfloop Research Ethics Committee (TREC) (No. (TREC/135/2023: IR).

## Results

### Analysis based on demographic data

A total of 371 SARS-CoV-2 samples were included in the study with a mean age of 44 (interquartile range [IQR], 31–57 years). The majority of the samples were from the 30–39 age group, with 72 (19%) samples while, age groups 80–89 and 0–9 age groups were the least frequent age groups with 9 (2.4%) and 8 (2.2%) samples respectively. Majority of the study samples, 208 (56 %) were females and only 3 (0.8%) had unknown gender.

### Positivity of the three antigen point-of-care tests kits

**FIGURE 1:**
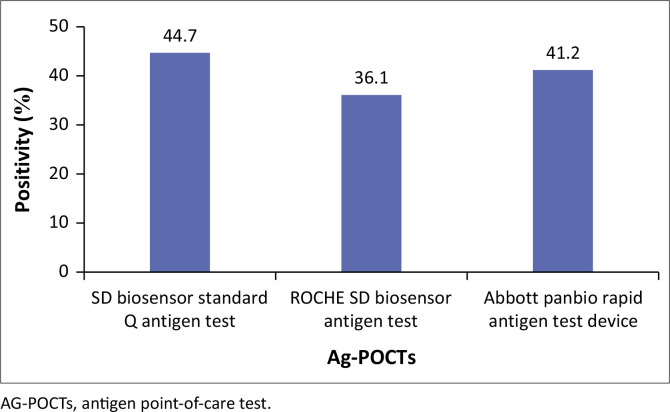
Proportion of positive severe acute respiratory syndrome coronavirus 2 samples detected per antigen point-of-care test (*N* = 371).

Of the 371 included nasopharyngeal swabs, the SD Biosensor Standard Q test kit detected the most positive isolates 166 (44.7%), followed by the Abbott Panbio Rapid Antigen test kit which detected 153 (41.2%) positives, while Roche SD Biosensor kit detected 134 (36.1%) of the samples (Figure 1).

**FIGURE 2:**
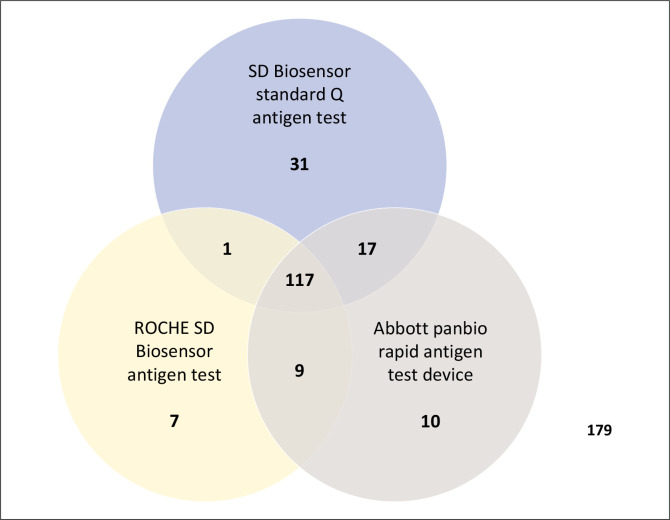
Overall severe acute respiratory syndrome coronavirus 2 samples detected per kit (*N* = 371).

The SD Biosensor Standard Q antigen tests detected the most samples which were not detected by other Ag POCT with 31 (8.4%). The SD Biosensor Roche RDT detected 7 (1.9%) while the Abbott Panbio Rapid Antigen Tests detected 10 (2.7%) not detected by other Ag POCT (Figure 2).

**FIGURE 3:**
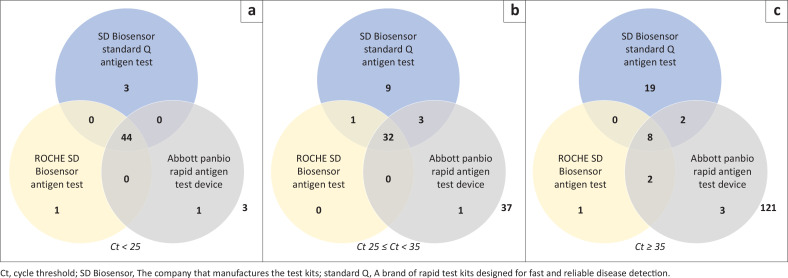
Venn diagram showing detected samples for the different cycle threshold values: (a) Ct < 25; (b) Ct 25 ≤ Ct < 35; (c) Ct ≥ 35.

Of the 52 samples with Ct < 25, the SD Biosensor Standard Q antigen test kit detected 47 (90.4%) positives, while the Roche SD Biosensor antigen test and Abbott Panbio Rapid Antigen test device detected 45 (86.5%) positive samples, respectively. Only 3 (5.8%) samples were negative on all the Ag-POCTs (Figure 3: Ct ≥ 35).

Of the 83 samples with 25 ≤ Ct < 35, the SD Biosensor Standard Q test kit detected 45 (54.2%) positives, while the ROCHE SD Biosensor antigen test and Abbott Panbio Rapid Antigen test device detected 33 (39.8%) and 36 (43.4%) positive samples, respectively (Figure 3: 25 ≤ Ct < 35).

A total of 37 (44.6%) of the RT-PCR positive samples were negative on all the Ag-POCTs. Of the 156 samples with Ct ≥ 35, SD Biosensor Standard Q detected 29 (18.6%), while ROCHE SD Biosensor antigen test and Abbott Panbio Rapid Antigen test device detected 11 (7.1%) and 15 (9.6%). A total of 121 (77.6%) samples in this category were negative on all the Ag-POCTs (Figure 3: Ct ≥ 35).

### Reverse transcription polymerase chain reactioncycle threshold values analysis

**FIGURE 4:**
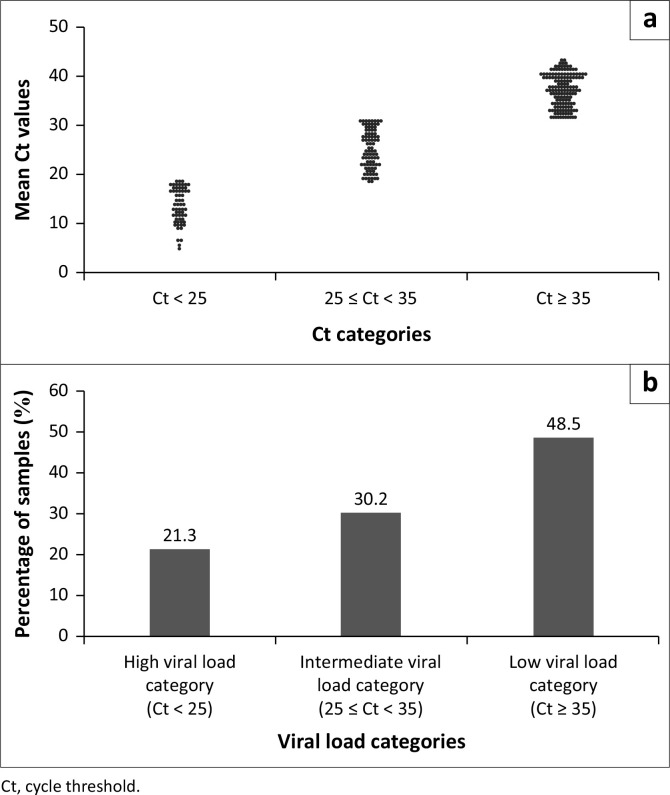
Reverse transcription polymerase chain reaction cycle threshold value ranges (*N* = 291). a) Ct categories; b) Viral load categories.

Of the 371 samples included, 291 (78.4%) were RT-PCR positive. The RT-PCR positive samples demonstrated viral cycle threshold (Ct) values ranging from 14 to 45. The mean Ct values were found to be 34 (IQR 27–40) and the mode for the dataset was 39, 5. The majority of the samples consisted of higher Ct values belonging to more than Ct > 36 (Figure 4).

### Performance of antigen point-of-care tests in samples with cycle threshold < 25 to cycle threshold < 35

**FIGURE 5:**
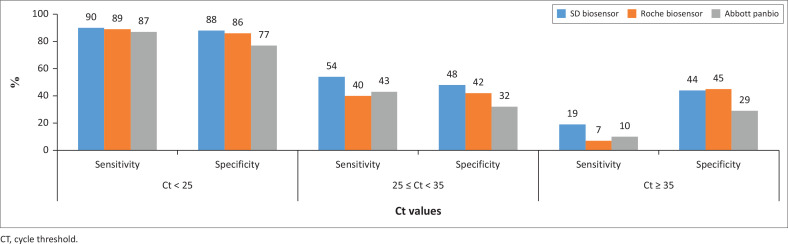
Sensitivity and specificity of the antigen point of care assays at different cycle threshold values.

For a high viral load of Ct < 25, there was high sensitivity of all Ag-POCTs tests which was found to be high above 77%. For intermediate Ct, the specificity and sensitivity were found to be a little over 32%. For samples with low viral loads the specificity was found to be as low as 29%, while sensitivity was found to be as low as 7% (Figure 5).

**FIGURE 6:**
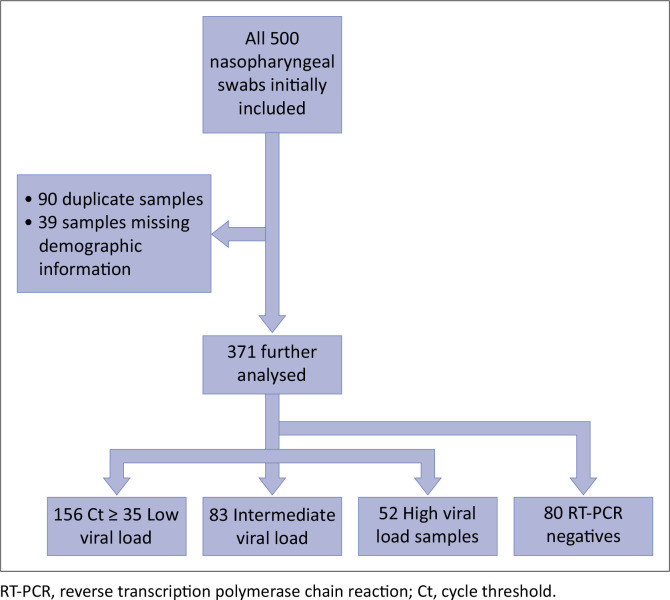
Classification of samples.

## Discussion

### Summary of key findings

The study evaluated the performance of three World Health Organization (WHO)-certified Ag-POCTs against the gold-standard RT-PCR technique in detecting SARS-CoV-2 across samples with varying viral loads. It found that the overall performance of the Ag-POCTs was suboptimal, especially in samples with low viral loads (Ct ≥ 35), where sensitivity dropped to as low as 7% and specificity to 29%. In comparison, samples with high viral loads (Ct < 25) showed better performance, with sensitivity and specificity above 77%. The SD Biosensor Standard Q test kit demonstrated the highest detection rate (44.7%) among the three, followed by Abbott Panbio (41.2%) and Roche SD Biosensor (36.1%).

The study highlights a significant discrepancy between the manufacturer’s reported sensitivity and specificity and the actual performance in clinical settings, particularly for low viral load samples. This suggests that Ag-POCTs are not reliable for diagnosing COVID-19 in the early or recovery phases of infection when viral loads are low. Consequently, the study recommends that rapid antigen tests should not be used as standalone diagnostics, especially in cases with low viral loads, and that RT-PCR should be requested to confirm negative Ag-POCT results. These findings are crucial for improving diagnostic accuracy and managing future pandemics in resource-limited settings.

### Discussion of key findings

As the healthcare system becomes more patient-centred, there is a rise in global demand for easy-to-use diagnostic, monitoring and screening assays.^11^ The use of Ag-POCTs has enhanced the capacity of healthcare systems to quickly identify and manage SARS-CoV-2 patients which has ultimately resulted in testing on a large-scale testing and reaching remote locations where laboratory-based NAATs would not be practical.^12^ Most healthcare facilities in Africa use point-of-care tests; however, some clinicians have expressed concern regarding the accuracy and reliability of the point-of-care tests, in the light of an increase in patients being misdiagnosed creating confusion among clinicians.^10^ This study is crucial for preparing accurate diagnostic assays for future infectious disease outbreaks.

In this study, the majority of the samples 156 (53.6%) belonged to the low viral load category (Ct ≥ 35). The current results agreed with post-factor COVID-19 pandemic studies that have also shown that most cases during the pandemic had low viral load.^13,14^ In one study, involving the Ct values of real-time RT-PCR, only 7% of the samples had high Ct values, 9% had moderate Ct values, and the remaining 84% had low viral loads.^13^ Thus, it is critical to find assays that can accurately detect samples with low viral load. The presence of the majority of samples having low viral load may be from the pandemic transgressing through various stages of progression. Pandemics may eventually assume low Ct values and/or milder symptoms, which may not necessarily translate to lower infection. Several factors may contribute to this observation which include mutations in the genome, environmental adaptions, and early or late onset of infections.^14^ Hence, previous studies have demonstrated that the rate of false-positive results may be high during the first 5 days after infection onset.^15^

This study assessed the performance of each of the three Ag-POCTs tested in the study against the gold standard test for SARS-CoV-2 diagnosis, RT-PCR assay. The results of this study have shown that Ag-POCTs had a lower-than-expected performance on clinical patient samples despite manufacturer and laboratory-based studies showing good accuracy in internal validations.

For the SD Biosensor Standard Q Antigen Test when tested against the high viral load samples category, the Ag-POCT we determined sensitivity and specificity of 90% and 88%, respectively. While in the low viral load category, the study determined sensitivity and specificity of 19% and 44%, respectively. The specificity and sensitivity were found to be very low compared to what manufacturer-reported sensitivity and specificity of 96.52% and 99.68% respectively according to the package insert. Previous studies have also reported comparatively very high sensitivity and specificity of above 90%.^16^

Whereas for ROCHE Biosensor Antigen, the current study determined the sensitivity and specificity of 89% and 86%, respectively in high viral load. While, in the low viral load category, we determined sensitivity and specificity of 7% and 45%, respectively. The specificity and sensitivity were found to be very low compared to the manufacturer reported. The package inserts report sensitivity and specificity of 97.6%, 91.8 and 93.3% at high Ct < 33 and even better performance at low Ct.

While the Abbott Panbio Rapid Antigen Test Device determined the sensitivity and specificity to be 87% and 77%, respectively.^17^ While in the low viral load (Ct > 35) category, we determined sensitivity and specificity of 10% and 29%, respectively. The specificity and sensitivity for Ct > 32 were found to be very low compared to manufacturer-reported sensitivity and specificity to be 93.8% and 100% respectively.^17^ Previous clinical evaluations showed a sensitivity below 75%^18^ or below 50% in some cases.^19^

The study reports general low sensitivity and specificity of antigen point-of-care tests when tested against low viral load samples. The results from the current study were comparatively lower than the manufacturer package insert from previously published results. The very high performance of package inserts, and previous studies may have been because of using pre-selected samples with high viral load and the possibility of repeating studies. Repeating study results is possible in laboratory settings but much harder and impractical in clinical settings, especially in areas of high movement and turnovers such as emergency department settings and clinical settings.

According to the WHO report, the average acceptable minimum performance of RDTs has been set at > 80% sensitivity and 97% specificity regarding symptomatic people.^20^. This study included only symptomatic patients whose samples have been referred to the laboratory after clinicians have confirmed COVID-19-related symptoms. The results of current studies show that the performance of the kits was well below acceptable rates in samples with low viral load.

These findings provide evidence and confirmation that the use of Ag-POCTs may not be recommended for diagnosis of SARS-CoV-2 infection particularly in phases of low viral shedding such as the incubation and convalescence courses of the infection, marked with predominantly low viral load in collected clinical specimens. The results have consequences that need to be considered for current and future pandemics. False-negative results can cause inadequate placement of patients in hospitals (e.g., moving an infectious patient to a ‘green ward’), causing new outbreaks in an already diseased population and also deem a community patient not infectious, thus increasing the risks of propagating infection to contacts. False positive results can inversely place patients in high-risk environments in hospitals (e.g., inside a ‘red ward’) and cause unnecessary isolation and economic impact in an outpatient setting. The study reports general low sensitivity and specificity of antigen point-of-care tests when tested against low viral load samples. The results of the current study were comparatively lower than those reported in the manufacturer’s package insert and previously published results.

### Strengths and limitations

Firstly, this study provided on SARS-CoV-2 detection on samples with low, intermediate and high viral load categories, specifically addressing samples with varying viral loads. This study has provided a guide to the laboratory-based techniques’ reviewers in the health care sector because currently in hospital settings of Limpopo Province SARS-CoV-2 testing is performed using Ag-POCTs. The focus on Limpopo Province, a low-resource setting, highlights the practical challenges and limitations of using Ag-POCTs in such areas.

The study primarily used remnant samples which were stored throughout the pandemic era; thus, the unavailability of clinical data about infection symptoms and the disease stages of the participants posed as a limitation. In addition, factors such as improper swabbing during sample collection and specimen transportation to the RT-PCR reference laboratory and storage of samples could have decreased the integrity of the specimens. Secondly, the study was conducted when COVID-19 restrictions were still in place in most hospital wards prohibiting contact with patients. Thus, the study was limited to being a laboratory study. Lastly, we did not collect information on co-morbidities and risk factors for SARS-CoV-2 diseases such as diabetes, HIV status, obesity, and socioeconomic status which are associated with SARS-CoV-2 seropositivity in other studies in South Africa.

### Implications

The study reported generally poor test performance of Ag-POCTs when testing nasopharyngeal swab samples with low viral load. Of the three Ag-POCTs, the SD Biosensor Standard Q antigen test showed greater sensitivity of detection and thus gave more reliable results. The study found the performance of Ag-POCTs tests were markedly different from the manufacturer’s reported performance. Therefore, this study suggests that rapid antigen tests should not be used as a standalone test for the correct diagnosis of infections, particularly in samples with low viral load. The study thus recommends clinicians to request for RT-PCR results in cases where the antigen tests results are negative.

## Conclusion

In conclusion, the study reported generally poor test performance of Ag-POCTs when testing samples with low viral load. The study found that Ag-POCT performed poorly in detecting SARS-CoV-2 in samples with low viral loads. Sensitivity was as low as 7%, although the Ag-POCT was more reliable in high viral loads, achieving 77% sensitivity. This highlights the unreliability of Ag-POCTS for diagnosing infections with low viral loads, thus necessitating RT-PCR confirmation. In addition, there was a significant performance gap between Ag-POCT and RT-PCR, particularly in samples with low viral loads, implying that Ag-POCTs could not be used as replacement for RT-PCR. In resource-constrained settings, where Ag-POCT are widely used, RT-PCR is essential to confirm negative results for accurate diagnosis and infection control.
